# Dynamics in Cardiometabolic Risk among Turkish Adults: Similarities to that in Iranians?

**Published:** 2011

**Authors:** Altan Onat

**Affiliations:** 1 Emeritus Professor, Cardiology Department, Cerrahpaşa Medical Faculty, Istanbul University, Istanbul, Turkey. Email: alt_onat@yahoo.com.tr

**Keywords:** Cardiometabolic risk factor, Ethnicity, Prediction, Prevention

## Abstract

Based on 20 years experiences of follow-up of the Turkish Adult Risk Factor (TARF) study, this review summarizes the distribution of risk factors among Turks which is dominated by components of the metabolic syndrome (MetS), especially abdominal obesity and atherogenic dyslipidemia. The adoption of a 95 cm cutoff for male abdominal circumference was crucial in the understanding of cardiometabolic risk factors. The prevalence of MetS, type-2 diabetes and coronary heart disease (CHD) are high, alike in Iranians. The TARF study demonstrated that low-grade systemic inflammation and oxidative stress are major determinants of cardiometabolic risk in the population at large, and involves the female sex to a greater extent than the male. As a result, impaired anti-inflammatory and atheroprotective function developed in middle-aged and elderly obese individuals emerging as dysfunction of apolipoprotein A-I and HDL particles. This dysfunction is currently a major driver cardiometabolic risk in Turkish adults leading to substantial excess diabetes and CHD. Separate algorithms for diabetes and CHD were derived that improved the risk prediction of these diseases.

The author strongly suspects that such dynamics in the development of diabetes and CHD exist in Western adults prone to impaired glucose tolerance, and evidence is accumulating regarding general Iranian adults. These issues posing a vast threat on public cardiometabolic health will have to be recognized with the purpose of not delaying implementation of measures for the modification of cardiometabolic risk, especially in women.

## INTRODUCTION

Edition of this review coincides with 20 years experience in the follow-up of a representative sample of Turkish adults which has revealed different aspects of cardiovascular disease (CVD) risk factors, metabolic syndrome (MetS), type-2 diabetes and coronary heart disease (CHD). This activity has generated new knowledge pertaining to the prevailing mechanisms of diabetes and CHD in the setting of population sections harboring proneness to impaired glucose tolerance which partly diverges from or adds to the available classical knowledge on CVD risk factors and pathogenesis. This information may be extremely attributable to Iranian adults since I suspect that several features of cardiometabolic risks are common in both populations. Ethnicity is a major determinant of central obesity, as is recognized by the guidelines of the International Diabetes Federation,^1^ and the former is a major determinant of cardiometabolic risks of most populations.

The Turkish Adult Risk Factor (TARF) study has been a longitudinal population-based cohort study on the prevalence of cardiac disease and its risk factors in Turkish adults carried out biennially since 1990 in 59 communities across geographical regions of the country.^2^ Participants, initially 3687 ones, are a random sample of the Turkish adult population aged 20 years or more, stratified for sex, age, geographical regions and rural-urban distribution. Data were obtained through past medical history via a questionnaire, physical examination of the cardiovascular system, blood samples and a resting 12-lead electrocardiogram.

## DISTRIBUTION OF RISK FACTORS AMONG TURKS AND SIMILARITIES TO IRANIAN ADULTS

In a cohort study, participants (mean age:49 ±12 years) displayed relatively large mean waist girths (94 and 90 cm in men and women, respectively), despite a non-proportionately high body mass index (BMI) in men (26.4 kg/m^2^), high fasting serum triglycerides (geometric means 152 and 136 mg/dl) and low HDL-cholesterol (38 and 45 mg/dl), while LDL-cholesterol was not elevated (114 and 119 mg/dl).^3^ Log C-reactive protein (CRP) levels averaged 1.96 mg/L and 2.2 mg/L in men and women, respectively. Fasting glucose and geometric insulin had a mean of 93 mg/dl and 7.7 mIU/L, respectively.^4^ Blood pressure in women (mean 132/83 mmHg) was 5/2 mmHg higher than men. Fifty four percent of men and 19% of women were current smokers, and 35% of men and 4% of women were alcohol drinkers. Low levels of HDL-cholesterol in Turks were first described in the Turkish Heart Study.^5^

These levels are essentially similar to non-diabetic Iranians of the same age,^6^,^7^ especially regarding low levels of HDL-cholesterol, with the following exceptions. Abdominal obesity and high triglyceride level in women, the major risk factors, seem to be less significant than blood pressure in Iranians. Total (and LDL-) cholesterol values were lower and current smoking was more prevalent among Turks of both genders, suggesting a higher prevalence of (familial) combined hyperlipidemia in Iranian adults.

## PREVALENCE OF METABOLIC SYNDROME, DIABETES AND CHD

In the past 6 years, the National Heart, Lung, and Blood Institute/American Heart Association definition of metabolic syndrome (Mets) have been modified^8^ using ≥95 cm cutoff for male abdominal obesity^9^ because one-third of Turkish men with high cardiometabolic risk were not comprised with the cutoff of 102 cm. Using this definition, MetS prevails in 41% of middle-aged and elderly men and 40% of women.^10^ The high rate of MetS in women is clearly lower than the 61% of MetS prevalence among Iranian females; and it is likely that the 34% prevalence in Iranian men is underestimated due to usage of a strict criterion for abdominal obesity, caused the high proportion of dysglycemia and low HDL-cholesterol in men “without” MetS.^11^ Moreover, even healthy Iranian adolescent were reported to show positive associations between a relatively low waist circumference, CRP and oxidative stress markers.^12^

The prevalence of type-2 diabetes, using the same definition^13^, reported to be 19% prevalent among Iranians,^11^ is also lower among Turks with 11% in the population aged 35 years.^14^ The incidence of type-2 diabetes is 11.6 per 1000 persons/year and rising. Prediabetes prevails among Turks also at a lower rate, namely 14% compared with the 27% in Iranian adults.^11^

Prevalence estimates of CHD are less reliable to compare. Nonetheless, we estimate that in the same age group, this prevalence is around 14% and the CHD incidence is 17 per 1000 person-years in Turkey. Considering the CHD incidence of 11 per 1000 person-years in Iranians,^11^ it may be 1.5-fold higher among Turks than Iranians. Alternatively, the incidence estimate for Iran may not include chronic CHD with (true) stable angina pectoris which is highly prevalent especially among females.

## GENDER DIFFERENCE

Both insulin resistance and CHD, particularly myocardial infarction, are recognized to have a greater predilection to the male sex. Yet, MetS, type-2 diabetes and CHD are virtually as common among Turkish women as men; and the coronary mortality in the age bracket 45-74 years discloses a smaller difference in the sexes as one would anticipate to. Hence, the protection afforded by female sex hormones appears to attenuate rapidly in the pre-menopausal and menopausal periods; and the increasing problems with overweight and obesity associated with high triglyceride and hypertension imposes a greater likelihood of cardiometabolic diseases. Investigators of the TARF study have come to learn that systemic low-grade inflammation are oxidative stress were major determinants of cardiometabolic risk, and involved female to a greater extent than male.

Other major gender differences are attributable to the prevalence of hypertension, the type of adiposity relevant to cardiometabolic risk and the pathway towards the development of CHD. As among Iranians, but to a greater extent, Turkish women have a higher prevalence of hypertension than age-matched men. This is not so, for instance, among Germans.^15^ Visceral adiposity in men and body fat in women are of greater relevance to cardiometabolic risk. Sex difference may reflect the predominating role of visceral adiposity in men and the pro-inflammatory state in women.^16^ While women go on from obesity (via the associated pro-inflammatory state) directly to diabetes, abdominally obese men with MetS develop diabetes.^17^ It is worth to search whether similar gender differences also exist in Iranian people, to have a more targeted approach to cardiovascular risk prevention.

## LOW-GRADE INFLAMMATION AND OXIDATIVE STRESS

It was recently reported that increased serum levels of CRP existed among Iranian women with raised waist circumference and elevated triglyceride.^18^ We have documented in the TARF study that low-grade systemic inflammation and oxidative stress were determinants of diabetes and CHD, independent of waist circumference, fasting glucose and HOMA in women though not in men.^19^ A 3-fold increment in multivariably adjusted CRP levels would predict the development of diabetes. When complement C3 levels, as markers of long-standing complement activation, were measured in order to predict diabetes or CHD, these were not independent determinants of the associated “pro-inflammatory state” in women or in men without MetS, but were independent determinants in men suffering MetS suggesting that C3 was a risk factor for both diabetes and CHD, additively to MetS in males.^20^,^21^

Serum gamma-glutamyl transferase (GGT) is known to reflect oxidative stress. According to the prospective analyses of 1667 adults at 4 year’s follow-up, we have found that elevated serum GGT confers, additively to BMI, risk of hypertension, MetS and type-2 diabetes but only mediates adiposity against CHD risk (unpublished observations). Each 1-SD increment in GGT activity would predict these metabolic disorders in each sex with hazard ratios of 1.2 to 1.3. In studied Iranian adolescent, Kelishadi et al.^22^ found that CRP, markers of oxidative stress and insulin resistance were affected independently by air pollutants, in addition to dietary habit.

## DYSFUNCTION OF APOLIPOPROTEIN A-I AND HDL PARTICLES

We have found that increased serum concentrations of apolipoprotein (apo) A-I, with anti-inflammatory and atheroprotective activities, rather than protection against cardiometabolic risk, would raise the risk of type-2 diabetes by 1.8-fold in each sex.^23^ We later demonstrated that the positive association exist in women was independent of HDL-cholesterol level (3) as well as of apoE genotype and apoB levels.^24^ We hypothesize that the protective activity of apoA-I may impair involve in auto-immune mechanisms; indeed, apoA-I has recently been reported to be combined during oxidation to LDL (apoAI-LDL), high levels of which could mark in a cross-sectional study on coronary artery disease more accurately than CRP.^25^

Low HDL-cholesterol level is a major cardiovascular risk factor and each 1 mg/dl increase in HDL-cholesterol was estimated to be associated with 2-3% decrease in the multi-adjusted risk of CHD. This may not apply universally, particularly in individuals prone to impaired glucose tolerance (reviewed in Ref. 17), it has been shown for the first time in a general population of Turks.^3^ Cox regression analyses presented in Table 1 indicate that HDL-cholesterol does not afford protection against CHD among women and cause modest protection in men implicating impaired function.

Presence of a similar dysfunction of HDL-cholesterol has been suggested in a recent report among Iranian women.^6^ Furthermore, sex-adjusted result of the TLG study showed that the prevalence of low HDL-cholesterol was far lower in people free of MetS, prediabetes or diabetes mellitus, strongly suggesting that higher HDL-cholesterol levels are heterogeneous and often associated with impaired glucose metabolism.

## PREDICTORS OF METS, DIABETES AND CHD

Regarding the predictors and nature of the MetS, the threshold of one of its components, abdominal obesity, depends largely on ethnicity. People prone to insulin resistance or IGT and Far Eastern populations are known to harbor a lower threshold. Beyond this we found that acute phase proteins/ inflammatory biomarkers such as CRP,^19^ complement C3^20^ and fibrinogen^26^ were independent determinants of MetS. The independency of the latter two markers from waist circumference, after adjusted for MetS or stratified in subjects with MetS, was largely confined to men because these risk factors, which were inherently defined as strong determinants of cardiometabolic risk, acted conjointly rather than in an independent manner in Turkish women. CRP, however, appears to act independently, especially in diabetics, in women as well.^19^

Regarding the development of type-2 diabetes, elevated circulating apo C-III on HDL particles was a strong determinant.^27^ Complement C3 proved to be a risk factor in Turkish men with newly developing CHD, additively to age, presence of MetS and lipid-lowering medication.

## ROLE OF CIGARETTE SMOKING

Though in not all but some ethnicities, cigarette smoking reduced the risk of the development of (abdominal) obesity. This was shown prospectively in Turks in both genders.^8^ Moreover, in linear regression analyses we have found an inverse association between current smoking and plasma C3 elevation in both genders independent of confounders such as waist circumference and serum triglycerides.^20^ Comparing other risk factors such as lower fasting insulin levels in current compared with never smokers, these factors may largely account for the protective effect of smoking in Turkish adults, particularly women, against MetS and type-2 diabetes mellitus (DM).^28^

Lower prevalence of ever smoking in dysglicemic Iranians without MetS comparing subjects with normal glucose regulation^11^ might reflect the effect of current smoking similar to Turks.

## ALGORITHMS FOR DM AND CHD

The prominent role of enhanced pro-inflammatory factors associated with dysfunction of HDL and apoA-I particles among middle-aged Turks prompted us to design specific appropriate algorithms in Turks that incorporated CRP as an inflammatory biomarker for prediction of type-2 diabetes and CHD. Before selecting the best fitting model, we adopted a backward strategy and analyzed the complete sample with a large number of variables. Male sex, family history of DM, impaired fasting glucose and raised waist circumference were predictors of diabetes in both sexes, while non HDL-cholesterol, (at borderline significance) age in men and physical inactivity were effective only in women. CRP would predict diabetes with borderline significance in men, whereas in women risk of diabetes would increase significantly with incremental CRP categories and had greater predictive value than fasting glucose level at baseline. Age, current smoking and non HDL-cholesterol were not significant factors in women.^29^ The final risk prediction algorithm is shown in table 1.^29^ A total of 8 factors in males and 10 ones in females will predict 20% or higher probability of developing diabetes over the coming 10 years.

**Figure 1:**
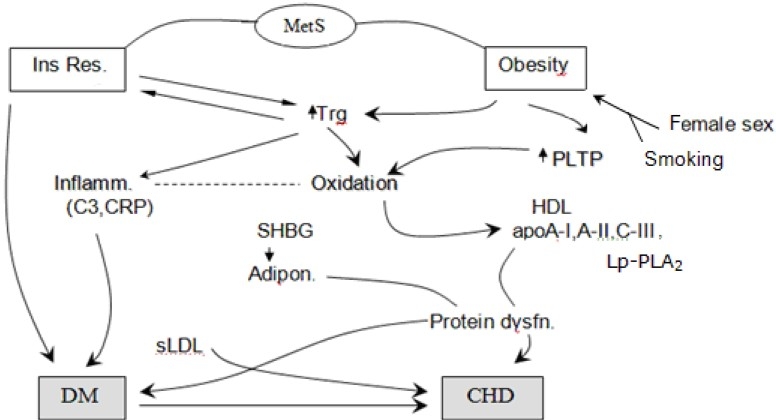
(reproduced from ref. 21). Pathogenetic factors involved in enhanced cardiiometabolic risk in a population with a high prevalence of MetS. The central roles of low-grade inflammation/oxidation and dysfunctions of HDL, its apoproteins and adiponectin and the potential influence of elevated circulating phospholipids transfer protein (PLTP) as well as of sex and sex hormone-binding globulin (SHBG) are illustrated. CHD, coronary heart disease; CRP, C-reactive protein; DM, type-2 diabetes; Ins Res., insulin resistance; Lp-PLA2, lipoprotein-associated phospholipase A2; sLDL, small LDL particles; Trg, triglycerides.

**Table 1:** Algorithm for prediction of diabetes risk among Turks 29

		Male	Female
Family history of diabetes	Yes	3	2
Physical activity	Yes	-1	-2
Age, yrs	41-50 y	1	0
	51-59 y	3	1
	≥60 y	2	0
Waist circumference, cm	93-103/ 88-101 cm	1	3
	≥104/102 cm	3	5
Fasting glucose, mmol/L	>5.55	3	2
C-reactive protein, mg/L	Male >0.8	2	
	Female 0.81-6.3		3
	≥6.3		5
Non HDL-cholesterol, mmol/L	3.63-4.32	2	0
	≥4.32	2	1

Reference categories receive no points.

Estimation of CHD risk was not affected by smoking status, HDL- and LDL-cholesterol levels in women (Table 2).^30^ Age, presence of diabetes, high systolic blood pressure and C-reactive protein (CRP) were predictors in both sexes (it is mentioned in previous sentence). AROC of the model was 0.789 in men, 0.806 in women (p<0.001). An algorithm using the stated 7 variables, designed separately for each sex, is shown in Table 3.^30^ A total of 10 factors in males and 11 ones in females will predict a 20% or more probability of developing CHD over a 10-year period.

These algorithms urge the need for populations prone to MetS to derive algorithms on own population rather than adopting the Framingham risk factors, even after recalibration.

**Table 2:** Cox regression analysis of risk factors for incident CHD in future (modified from 30)

	β	HR	95%CI	β	HR	95%CI
	
	Men, n=158/1043*			Women, n=144/1189		
LDL-cholesterol ≥130 mg/dL	0.725	**2.06**	1.38;3.09	0.358	1.43	0.87;2.35
Current vs. non-smokers	0.452	**1.57**	1.06;2.32	-0.183	0.83	0.50;1.38
HDL-chol.† 40-49/50-59 mg/dl	0.474	1.61	0.85;3.03	0.242	1.27	0.72;2.26
<40/<50 mg/dl	0.57	*1.77*	1.00;3.13	0.158	1.17	0.66;2.08
CRP¶, mg/L† 2.8-4.99/ 3.21-6.3	0.674	**1.96**	1.16;3.31	0.756	*2.03*	0.97;4.25
>5/ >6.3	0.485	*1.62*	0.98;2.68	0.953	**2.59**	1.25;5.38

* incident CHD/ number at risk. CRP, C-reactive protein

† categories are sex-specific.

Also included in Cox models were age, presence of type-2 diabetes and systolic BP. Reference categories were: LDL-C <100 mg/dl, non-smoker, HDL-C ≥50/60 mg/dL, CRP <0.8 mg/L.

**Table 3:** Algorithm for predicting incident CHD risk among Turkish men and women, aged 30-74 yrs (30)

Criterion	Category	Men	Women
Age, yrs	40-49	4	4
	50-59	5	8
	≥60	8	10
Presence of diabetes	Yes	2	3
LDL-cholesterol, mg/dL	≥130	2	1
Systolic BP, mmHg	120-139	1	2
	140-159	2	2
	≥160	4	4
Current vs. non-smoker	Yes	2	0
HDL-cholesterol, mg/dL	≥50/60	0	0
	40-49/50-59	0	0
	<40/<50	2	0
C-reactive protein, mg/L	M≥3.0; F 0.8-6.3	2	2
	>6.3	2	3

Reference categories receive no points: age 30-39 years, nondiabetic, LDL-C <130 mg/dl, SBP <120 mmHg, nonsmoker, HDL-C ≥50/60 mg/dL, CRP <3 in males, <0.8 mg/L in females.

## CARDIOVASCULAR PREVENTION IN TURKEY

The risk factor constellation in Turkey outlined in this review suggests that (abdominal) obesity, diabetes and hypertension are of paramount importance in cardiovascular prevention, while serum LDL-cholesterol and, in women, cigarette smoking are of lesser significance, a statement changing compared to guidelines for global cardiovascular prevention.

The National Heart Health Policy of the Turkish Ministry of Health established priorities at 2006.^31^ The priority afforded overemphasis on cigarette smoking. Increased public awareness for treating hypertension was achieved, the prevalence of which has been declining since year 1998, due to a rapid rise in widespread use of antihypertensive drugs. Another major focus on heart failure seems not well-placed in Turkey – having far fewer elderly people- compared with communities in developed countries.

Little effort is given for reversing the tide of diabetes and MetS which pose a huge public health problem. Even a sorely minimum standard of male abdominal obesity was not adopted.

Dysfunctions of HDL, apoA-I and other inflammatory mediators, obviously are not incorporated in the Framingham model, have a great or even greater impact than traditional risk factors on CHD events in this population. This explains why Turks have the top position in the ranking of coronary mortality in Europe in the age-bracket 45-74 years. It is uncertain what can be expected from high-profile ‘Care for Your Heart’ campaigns.

Refraining from recognizing a vast threat on cardiometabolic public health only will delay the improvement of cardiometabolic health of middle-aged and elderly Turks, especially women.
